# Hypophysitis Induced by Sintilimab in the Treatment of Bladder Cancer: A Case Report

**DOI:** 10.2174/0118715303257557231002064417

**Published:** 2024-03-26

**Authors:** Ran Li, Baichuan Jiang, Yiran Zhu, Likuan Gao, Yaru Zhou, Shijie Yang

**Affiliations:** 1 Department of Urology, The Third Affiliated Hospital of Hebei Medical University, Shijiazhuang, China;; 2 Department of Endocrinology, The Third Affiliated Hospital of Hebei Medical University, Shijiazhuang, China

**Keywords:** Immune checkpoint inhibitor, sintilimab, immune-related adverse events, hypophysitis, isolated ACTH deficiency, anorexia, drowsiness

## Abstract

**Background:**

Immune checkpoint inhibitors (ICIs), as novel antitumor drugs, have been widely used in the clinic and have shown good antitumor effects. However, their widespread use has also led to the emergence of various immune-related adverse events (IrAEs). Hypophysitis is a rare but serious IrAE. Due to its complex and changeable clinical manifestations, hypophysitis may be easily overlooked, leading to delayed diagnosis and treatment.

**Case Presentation:**

A 68-year-old male patient was diagnosed with bladder cancer (T_2b_N_X_M_0_) in October 2021. He received two cycles of immunotherapy with sintilimab and chemotherapy with gemcitabine and cisplatin (GC). One month after the second treatment, he gradually developed recurrent fever, anorexia, drowsiness, and delirium. Laboratory examination revealed hyponatremia, decreased adrenocorticotropic hormone, and hypocortisolemia. The pituitary MRI showed no abnormality. The patient was diagnosed with immunotherapy-induced hypophysitis (IH) caused by sintilimab, leading to downstream endocrine disorders. With hormone replacement therapy, he was in a good mood, had a good appetite, and made an overall recovery.

**Conclusion:**

Immunotherapy-induced hypophysitis (IH) can result in a severe adrenal crisis, and prompt recognition and diagnosis are crucial. Clinicians must remain vigilant for the possibility of IH in patients who exhibit recurrent fever, anorexia, cognitive decline, and personality changes following ICI treatment. It is imperative to consider this diagnosis early to initiate appropriate management promptly.

## 1 INTRODUCTION

Immune checkpoint inhibitors (ICIs) have emerged as a vital tool for anticancer therapy, exhibiting robust anti-tumor effects in clinical practice. ICIs include cytotoxic T-lymphocyte-associated antigen-4 (CTLA-4) inhibitors, programmed cell death protein-1 (PD-1) inhibitors, and programmed cell death ligand-1 (PD-L1) inhibitors, which restore the cytotoxicity of T cells against tumors by binding to immune checkpoint proteins on the surface of T cells [1]. While they show good efficacy, inevitably, many side effects occur, called immune-related adverse events (IrAEs). IrAEs can involve multiple systems, such as the endocrine, rheumatological, pulmonary, and neurological systems [2]. Immunotherapy-induced hypophysitis (IH) is one of the adverse events involving the endocrine system, which is characterized by an insidious onset and non-specific symptoms that can result in delayed diagnosis and treatment.

Here, we report a case of hypophysitis induced by sintilimab in the treatment of bladder cancer and summarize the clinical features of the resulting isolated ACTH deficiency and treatment strategy. The aim is to raise clinicians' awareness of IrAEs.

## 2 CASE REPORT

A 68-year-old Asian male was diagnosed with bladder cancer (T2bNXM0) in a local hospital on October 25, 2021, and received two cycles of immunotherapy with sintilimab and chemotherapy with gemcitabine and cisplatin (GC) without any adverse effects: Gemcitabine 1.4 g (d1), 2 g (d8) + cisplatin 30 mg (d1, d2, d3) in combination with sintilimab 200 mg (d1) were given on October 25, 2021, and gemcitabine 1.8 g (d1, d8) + cisplatin 30 mg (d1, d2, d3) in combination with sintilimab 200 mg (d1) were given on November 24, 2021. Before the treatment, his liver and kidney functions and blood pressure were normal, there were no endocrine and electrolyte disorders with him, and the patient's condition was stable during treatment. On December 31, 2021, the patient began to develop recurrent fevers (up to 39.0°C), for which he received antipyretic therapy (dexamethasone 10 mg IV) at the local hospital, resulting in fever control. He presented with laboratory findings indicative of hyponatremia (serum sodium: 121 mmol/L), which was successfully corrected with intravenous sodium supplementation. He was discharged from the hospital, while chemotherapy and immunotherapy were discontinued. However, on March 1, 2022, he was readmitted to the local hospital due to loss of appetite. Laboratory tests continued to show hyponatremia, prompting the administration of oral medication and intravenous sodium supplementation. Despite these interventions, symptoms persisted, and hyponatremia remained unresolved, leading to transfer to our hospital.

He had a 40-year history of smoking and alcohol consumption and exhibited poor spirits and bilateral foot edema upon physical examination. No suprapubic bladder elevation or significant pressure tenderness was observed, and the patient's Eastern Cooperative Oncology Group (ECOG) score was 4. Laboratory tests: Blood routine examination: white blood cell (WBC) was 6.71×10^9^ /L (reference value: 3.5-9.5×10^9^ /L); eosinophil (EOS #) was 0.96×10^9^ /L (0.24×10^9^ /L before sintilimab treatment; reference value: 0.02-0.52×10^9^ /L). Biochemical items: albumin (ALB) was 36 g/L (reference value: 40-55 g/L); sodium (Na+) was 127.78 mmol/L (reference value: 137-147 mmol/L); osmotic pressure (OSM) was 270.9 mOsm /L (reference value: 280-310 mOsm/L). The pelvic CT scan with contrast revealed a bladder mass involving both ureteral orifices. The above examinations indicated anemia, increased eosinophil, hypoproteinemia, hyponatremia, and malignant bladder tumors.

**Fig. (1):**
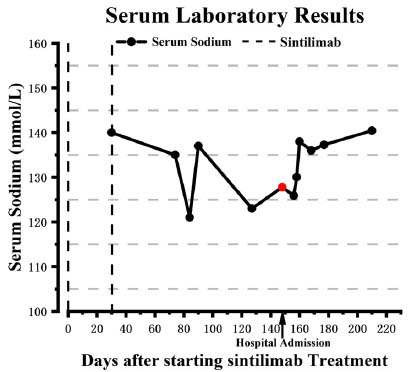
The patient’s serum sodium over time. Serum sodium remained normal when the patient received the second immunotherapy on the thirtieth day. After that, his serum sodium levels dropped and became low even after being corrected. After treatment at our hospital, his serum sodium rebounded and fluctuated within the normal range.

On the eighth day, the patient presented with a fever reaching a maximum of 39.3°C, which responded to treatment with antipyretics. But he experienced recurrent episodes of fever after that. Pathogen DNA testing identified Aspergillus as the causative agent, while the galactomannan (GM) test showed a value of 1.404 I (reference value: <0.5 I), suggesting a fungal infection. Prompt administration of voriconazole and allicin helped maintain his body temperature within the normal range. Additionally, intravenous sodium replacement and oral tolvaptan tablets were given, normalizing his serum sodium level (Fig. **1**). On the thirteenth day after admission, his serum sodium level was 138 mmol/L, fluctuating within the normal range thereafter.

**Fig. (2a, b):**
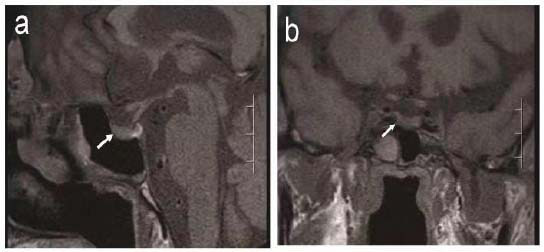
Magnetic resonance imaging (MRI) results. MRI showed that the pituitary gland was normal in size and morphology. Its upper edge was straight without protuberance, and there was no obvious abnormal signal shadow in it.

However, he exhibited a progressive decline in consciousness, accompanied by lethargy and impaired cognitive function, including memory, orientation, and calculation. Neurological evaluation revealed no significant abnormalities and muscle strength was graded at approximately IV. His blood pressure remained stable, and liver and kidney functions were within normal range. Cranial CT scans did not indicate any apparent central nervous system pathology. After a consultation with an endocrinologist, the hormone evaluation was screened, indicating reduced levels of ACTH and cortisol (Table **1**). The pituitary MRI (Fig. **2**) did not reveal any significant abnormalities. Intravenous hydrocortisone 50 mg q12h was administered immediately, resulting in improved sleep, consciousness, and appetite. After the patient stabilized, oral prednisone was substituted for intravenous hydrocortisone. Through monitoring cortisol levels, it was gradually adjusted to the lowest effective dose (5 mg every morning and 2.5 mg every night). One month after discharge, the patient returned to the clinic with improved mental status and appetite, an ECOG score of 1, no fever, and normal serum sodium and cortisol levels. But, his plasma ACTH level remained low at 2.921 pg/ml.

## 3 DISCUSSION

In this case, the patient had no prior endocrine dysfunction but presented with a range of symptoms after 68 days of treatment with sintilimab, initially manifested as recurrent fever, hyponatremia, loss of spirit and appetite, and then obstinate hyponatremia, impoverished spirit and appetite, drowsiness, and delirium, and accompanied by reduced cortisol and ACTH levels. The patient under consideration developed IH and secondary adrenocortical insufficiency. No other drugs were administered during treatment except for gemcitabine and cisplatin, and no endocrine-related adverse events were reported by checking gemcitabine and cisplatin.

Sintilimab is a fully humanized IgG4 monoclonal antibody that can bind with PD-1 and specifically block the interaction between PD-1 and its two known ligands, PD-L1 and PD-L2, thereby helping to restore the anti-tumor effect of T cells [3-5]. *In vitro* experiments, sintilimab demonstrated high affinity and specificity for human PD-1 and effectively inhibited the binding of human PD-1 to PD-L1 and PD-L2 [6]. ICIs can augment the risk of autoimmune toxicities, resulting in immune-related adverse events (IrAEs) [1, 2]. Dolladille reported that IrAEs are often severe, with some being fatal [7]. IrAEs can affect almost all systems and organs of the body. For example, in the skin, they manifest as pruritus, rash, and vitiligo; in the lungs as pneumonia; in the endocrine system as hypothyroidism, hyperthyroidism, hypophysitis, adrenocortical hypofunction, diabetes, *etc*. [2, 8]. IH induced by PD-1 inhibitor is often characterized by isolated ACTH deficiency (IAD), which is a potentially fatal adverse reaction with decreased cortisol production [9, 10]. As per the Common Terminology Criteria for Adverse Events (CTCAE) published by the National Cancer Institute (NCI), the severity of IH is classified as grade 5 [11], and the patient's condition reached grade 4, highlighting the severe nature of IH and its potential impact on patient outcomes.

IH is a rare adverse event with variable onset time. The incidence of hypophysitis following PD-1 inhibitor treatment was reported to be 1% [12]. Retrospective analysis showed that the onset time of hypophysitis induced by PD-1/PD-L1 antibodies also varied widely, with some cases developing rapidly over several weeks and most cases developing after an average of 27 weeks, while a few cases occurred even after a year [3]. Therefore, the patients need long-term follow-up and dynamic monitoring after treatment with ICIs.

Up to now, the diagnosis of IH has primarily relied on the clinical presentation of pituitary hormone deficiency because of the lack of specific serological markers and clear MRI manifestations of the hypophysis. The hallmark endocrine abnormality is secondary adrenocortical insufficiency resulting from diminished ACTH secretion by the adenohypophysis, leading to reduced cortisol production and release from the adrenal glands. This may manifest as fatigue, anorexia, nausea and vomiting, fever of unknown causes, varying degrees of cognitive dysfunction, hypotension, hypoglycemia, obstinate hyponatremia, and other symptoms [13, 14]. In imaging manifestations, Studies have demonstrated that hypophysitis resulting from CTLA-4 blockade typically leads to panhypopituitarism with mild pituitary enlargement, while the PD-1 blockade group is characterized by isolated severe ACTH deficiency without mass effect symptoms and imaging abnormalities [3]. In terms of laboratory tests, in addition to measuring relevant hormone levels, adrenocortical stimulation tests using synthetic ACTH and CRH stimulation tests can also assist in the diagnosis. Eosinophilia has been noted as a potential early predictor of adrenocortical insufficiency [15], and this patient's blood eosinophil count was higher than normal. But, its relevance and predictive accuracy require further research and examination, as they can be influenced by various factors such as infection, drug reactions, and allergies. Therefore, we were unable to utilize the eosinophil count as an early predictor, which serves as a valuable lesson. Consequently, we propose the following diagnostic key points from this case: a history of previous ICI treatment, symptoms associated with adrenocortical insufficiency after using ICI, reduced levels of ACTH and cortisol, and MRI imaging of the pituitary gland with or without evident changes.

Management of IH is a significant concern for clinicians, given the potential for permanent adrenal axis damage [2, 16]. At present, the treatment mainly relies on steroids. The European Society of Medical Oncology (ESMO) issued a clinical practice guideline on toxicity management of immunotherapy in 2022, which recommended that hormone replacement therapy should be provided for multiple deficiencies when affected, most importantly cortisol deficiency [17]. Developing a treatment plan typically requires collaboration with an endocrinologist. Prednisone (or hydrocortisone) dosage is gradually adjusted based on thrice-daily cortisol measurements until the lowest effective dose is achieved. Patients should take the medication in divided doses, morning and evening, to maintain a balanced blood hormone level throughout the day. In addition, it is crucial to administer a high dose of glucocorticoid supplement during periods of stress, such as the perioperative period, to prevent adrenal crisis. After the patient recovered, we performed transurethral resection of the bladder tumor twice triumphantly. Our clinical practice has demonstrated that a large amount of glucocorticoid supplementation before, during, and after surgery is the cornerstone to ensure perioperative safety. However, we have observed a limitation in the current treatment approach, where although the patient’s serum cortisol levels remain normal, his serum ACTH levels do not return to normal levels.

## CONCLUSION

At present, the pathogenesis of IH remains unclear. Its diagnosis is challenging due to the lack of specific serum markers and imaging changes. Additionally, the rarity, insidious onset, nonspecific symptoms, and interference of confounding factors during diagnosis and treatment make it difficult to detect until patients exhibit severe clinical symptoms. Therefore, standardized diagnosis and treatment, careful consideration, and multidisciplinary consultation are essential for managing such patients. Clinicians should thoroughly understand the indications for ICIs, prioritize patient informed consent, ensure adequate follow-up, and possess a comprehensive knowledge of IrAEs and their management principles. In patients receiving ICI therapy, clinicians must be mindful of the possibility of IH if someone presents with recurrent fever, anorexia, refractory hyponatremia, or varying degrees of cognitive dysfunction and administer timely and accurate diagnosis and treatment to prevent adrenal crisis.

## Figures and Tables

**Table 1:** Comprehensive hormone screening for the patient.

**Hormone**	**Result**	**Reference Range**
Cortisol (0:00)	0.75	20-60 ng/ml
Cortisol (8:00)	0.78	60-230 ng/ml
Cortisol (16:00)	1.06	30-110 ng/ml
ACTH	2.471	6-48 pg/ml
LH	12.08	1.24-8.62 mIU/ml
FSH	25.96	1.27-19.26 mIU/ml
PRL	311.29	55.9-278.3 mIU/L
PROG	0.17	0.45-6.55 nmol/L
E2	<55.1	55.1-142.9 pmol/L
T	7.89	6.07-27.1 nmol/L

**Abbreviations:** ACTH: Adrenocorticotropic hormone, LH: luteinizing hormone, FSH: follicle-stimulating hormone, PRL: prolactin, PROG: progesterone, E2: estradiol, T: testosterone.

## LIST OF ABBREVIATIONS

ACTH: Adrenocorticotropic Hormone
GM: Galactomannan
IAD: Isolated ACTH Deficiency
ICIs: Immune Checkpoint Inhibitors
IH: Immunotherapy-induced Hypophysitis
IrAEs: Immune-related Adverse Events
MRI: Magnetic Resonance Imaging.
